# Impaired Differentiation of Langerhans Cells in the Murine Oral Epithelium Adjacent to Titanium Dental Implants

**DOI:** 10.3389/fimmu.2018.01712

**Published:** 2018-08-15

**Authors:** Oded Heyman, Noam Koren, Gabriel Mizraji, Tal Capucha, Sharon Wald, Maria Nassar, Yaara Tabib, Lior Shapira, Avi-Hai Hovav, Asaf Wilensky

**Affiliations:** ^1^Department of Periodontology, Faculty of Dental Medicine, Hebrew University—Hadassah Medical Center, Jerusalem, Israel; ^2^Faculty of Dental Medicine, The Institute of Dental Sciences, Hebrew University, Jerusalem, Israel

**Keywords:** dental implants, peri-implantitis, Langerhans cells, peri-implant epithelium, langerin

## Abstract

Peri-implantitis is a destructive inflammatory process affecting tissues surrounding dental implants and it is considered a new global health concern. Human studies have suggested that the frequencies of Langerhans cells (LCs), the main antigen-presenting cells (APCs) of the oral epithelium, are dysregulated around the implants. Since LCs play a role in regulating oral mucosal homeostasis, we studied the impact of dental titanium implants on LC differentiation using a novel murine model. We demonstrate that whereas the percentage of LC precursors (CD11c^+^MHCII^+^) increased in the peri-implant epithelium, the frequencies of LCs (CD11c^+^MHCII^+^EpCAM^+^langerin^+^) were significantly reduced. Instead, a population of partially developed LCs expressing CD11c^+^MHCII^+^EpCAM^+^ but not langerin evolved in the peri-implant mucosa, which was also accompanied by a considerable leukocyte infiltrate. In line with the increased levels of LC precursors, expression of CCL2 and CCL20, chemokines mediating their translocation to the epithelium, was elevated in the peri-implant epithelium. However, expression of TGF-β1, the major cytokine driving final differentiation of LCs, was reduced in the epithelium. Further analysis revealed that while the expression of the TGF-β1 canonical receptor activing-like kinase (ALK)5 was upregulated, expression of its non-canonical receptor ALK3 was decreased. Since titanium ions releasing from implants were proposed to alter APC function, we next analyzed the impact of such ions on TGF-β1-induced LC differentiation cultures. Concurring with the *in vivo* studies, the presence of titanium ions resulted in the generation of partially developed LCs that express CD11c^+^MHCII^+^EpCAM^+^ but failed to upregulate langerin expression. Collectively, these findings suggest that titanium dental implants have the capacity to impair the development of oral LCs and might subsequently dysregulate immunity in the peri-implant mucosa.

## Introduction

Dental implants provide a successful solution in replacement of missing teeth, with more than two million dental implants are placed annually around the world (1). The use of dental implants brought upon a new disease, peri-implantitis, which became a major health concern worldwide (2–8). A recent epidemiologic review reported that every fifth dental implant eventually develops peri-implantitis during a mean functional loading time of 3.4–11 years (8). Titanium implants are mostly used for dental rehabilitation; nevertheless, titanium micro-particles were found to be released into peri-implant mucosa and their presence was proposed to alter local mucosal immunity (9–16). Moreover, titanium ions were shown to alter the maturation and migration capabilities of dendritic cells (DCs) differentiated from human monocytes (11). It is thus important to reveal how titanium implants alter oral mucosal immunity, as such knowledge will increase our understanding of the unknown pathogenesis of this new disease together with our capacity to develop therapeutic strategies to prevent dental implant-associated diseases and ultimately failure of the implants.

Langerhans cells (LCs) are a unique type of antigen-presenting cells (APCs) exclusively located in stratified epithelia such as the skin epidermis and the oral mucosa epithelium (17–20). Besides their distinctive anatomical location, LCs can be identified based on their capacity to express high levels of langerin (CD207) and epithelial cell adhesion molecule (EpCAM/CD326). Nevertheless, unlike epidermal LCs that originate from embryonic precursors (21, 22), oral LCs develop at steady state from adult bone marrow (BM) precursors: pre-DCs and monocytes (23). It has been shown recently that differentiation of these two precursors into LCs involves sequential signaling at two separate anatomical locations (24). Upon extravasation from the circulation, LC precursors acquire a CD11c^+^MHCII^+^ phenotype and later on exposed to bone morphogenetic protein 7 (BMP7), which its expression is restricted to the lamina propria (LP). Interaction of BMP7 with activing-like kinase 3 (ALK3), upregulates the expression of E-cadherin, CCR2, and CCR6 on LC precursors enabling their translocation to the epithelium. Within the epithelium, the precursors are exposed to TGF-β1 that finalizes their differentiation, resulting in an upregulation of EpCAM and later on langerin. This activity of TGF-β1 is considered to involve signaling *via* its canonical receptor ALK5; nevertheless, TGF-β1/ALK3 signaling is also likely to play a role in terminal LC differentiation. Oral LCs can be divided into at least two subsets, CD103^+^LCs and CD11b^+^LCs, which might represent two functionally diverse populations (23). On this regard, LCs were shown to play a regulatory function on oral mucosal immunity at both steady state and inflammatory conditions. Inducible ablation of LCs was shown to alter oral mucosal immunological functions and to induce microbial dysbiosis. Interestingly, oral microbiota also affects LCs development as the absence or decrease in the microbiota resulted in a reduced frequencies of oral LCs, particularly CD103^+^LCs (24). In a setting of infection with the oral pathogen *Porphyromonas gingivalis*, LCs were shown to have a protective function, inducing T regulatory (Treg) cells that inhibit the development of bone destructing Th1 immunity (25).

Given the important role of LCs on oral mucosal immunity, it is very likely that these cells might orchestrate the peri-implant immunological status. It is also possible that the presence of the implant will have some effects on the neighboring LCs. Nevertheless, there is a dispute in the literature regarding the impact of titanium dental implants on oral LCs. Various studies examined LCs in the tissue around dental implants have reported either a reduction or, alternatively, no alteration in the frequencies of these cells (26–30). Whereas such contradicting results could be explained by a variation in tissue sampling, technical approach, or the markers used to identify LCs, they highlight the necessity of an experimental model allowing meticulous analysis of this important topic. Using an experimental murine model, this study was initiated to explore whether and how oral mucosal LCs are regulated by titanium dental implants.

## Materials and Methods

### Mice

BALB/c mice (4–5 week old) were purchased from Harlan (Jerusalem, Israel). All the animals were housed in ventilated cages at room temperature under a 16 h light and 8 h dark cycle and received distilled water and food *ad libitum*. All animal experimental procedures were reviewed and approved by the IACUC of the Hadassah—Hebrew University Medical Center.

### Antibodies and Reagents

The following fluorochrome-conjugated monoclonal antibodies and the corresponding isotype controls were purchased from BioLegend (San Diego, CA, USA): CD45.2 (104), I-A/I-E (M5/114.15.2), EpCAM (G8.8), CD11b (M1/70), CD11c (N418), Langerin (4C7), CD205 (NLDC-145), CD103 (2E7), Ly6C (HK1.4), Ly6g (1A8), CD3 (17A2), CD4 (GK1.5), FOXP3 (MF-14), and B220 (RA36B2). Propidium iodide solution was also purchased from BioLegend.

### Titanium Implants

Custom-made titanium implants (MIS Implants Technologies Ltd., Israel) were made from Ti6Al4V alloy and were acid etched. The implants length was 1.7 mm and the implant diameter was 0.7 mm at the coronal end and 0.2 mm at the apical end.

### Extractions and Implantation

In each experiment, the mice were randomly divided into two groups, implanted and non-implanted. Animals in the first group were designated to receive two titanium implants following the extraction of the left upper molars whereas the mice in the control non-implanted group did not undergo the above-mentioned procedures. In order to ensure minimum interference with the implant osseointegration, the antagonist teeth of the lower jaw were extracted in both groups in order to reduce occlusal masticatory forces. All mice were anesthetized prior to the surgical procedure with an intra-peritoneal injection of ketamine (50 mg/kg) and Xylazine (10 mg/kg). In addition, analgesia was provided by sub-cutaneous injection (Carprofen, 5 mg/ml, 100 µl) and the mice were maintained with sterile soft diet for 1 week. To allow proper wound and bone healing the implants were inserted 4 weeks after teeth extraction, in the sites correlating to the original locations of the first and second molars. Under general anesthesia and analgesia, the osteotomy sites were prepared and the implants were inserted with custom-made driver (MIS Implants Technologies Ltd., Israel) until the implant’s threads were completely covered by bone and resistance and stability were achieved. Mice were given antibiotics (5% enrofoloxacin in drinking water) and soft diet for 7 days following implant insertion.

### Isolation and Processing of Gingiva and Peri-Implant Mucosa Tissues

The mice were euthanized and a circumference of 1 mm of gingiva around teeth in non-implanted mice, or 1 mm of peri-implant mucosa in implanted mice was harvested and pooled from two to three mice so that a sufficient number of cells were obtained for flow cytometry analysis. These pooled tissues were considered as *n* = 1. Next, the samples were incubated with Dispase II solution (Godo Shusei, Japan) 2 mg/ml in PBS + 2% fetal calf serum (FCS), for 45 min at 37°C. The epithelium was separated by using forceps under a stereoscope, and later on was minced and treated with Collagenase type II (2 mg/ml; Worthington Biochemicals) and DNase I (1 mg/ml; Roche) solution in PBS + 2% FCS for 25 min at 37°C in a shaker bath. A total of 20 µl of 0.5 M EDTA per 2 ml sample was added to the digested tissues and incubated for an additional 10 min. The cells were washed, filtered with a 70-µM filter, and stained with antibodies as indicated in the text. The stained samples were run in the LSR II (BD Biosciences) flow cytometer and analyzed using FlowJo software (Tree Star).

### Immunofluorescence Staining

The maxillae were fixed overnight at 4°C in 4% paraformaldehyde/PBS solution, and then washed for 1 week in EDTA 0.5 M/PBS that was changed every other day. Carefully, the implants were taken out in counterclockwise motion without damaging the surrounding tissue. Next, the tissue was cryopreserved in 30% sucrose (overnight at 4°C), embedded in OCT, and cryosectioned into 10-μm-thick sections. The cross sections, as well as the separated epithelium, were washed three times in PBS, blocked in a blocking buffer (5% FCS, 0.1% Triton X-100 in PBS) for 1 h at room temperature, and incubated with primary antibodies: goat anti-langerin (E-17, Santa Cruz Biotechnology), rat anti-MHCII (M5/114.15.2, BioLegend), rabbit anti-TGF-β1 (ab92486 Abcam), and mouse anti-BMP-7 (ab54904 Abcam) overnight at 4°C. Following three washing steps in PBS, the samples were incubated with a secondary antibody: donkey anti-goat IgG, donkey anti-rat IgG, or donkey anti-rabbit IgG (Jackson ImmunoResearch) diluted 1:100 in blocking buffer for 1 h at RT, washed three times, stained with Hoechst, and mounted. Signals were visualized and digital images were obtained using an Olympus BX51 fluorescent microscope mounted with a DP72 (Olympus) camera. The color intensity was calculated using Image J on 8-bit images by countering the specific area of interest. The average pixel intensity was calculated from a pixel histogram representing the color intensity of each pixel. Negative control was achieved by staining the tissue with the same primary antibody using non-compatible secondary antibody.

### Picrosirius Red Staining

The cryo cross sections slides were washed once in PBS for 30 min, followed by staining with Weigert’s hematoxylin for 8 min. Immediately after, the slides were washed for 10 min in running tap water, followed by staining with Picrosirius red for 1 h in dark. The staining was washed in acidified water that was changed twice and dehydrated for 5 min each time in 100% ethanol that was replaced three times. The slides were then incubated with xylene for 5 min followed by mounting in resinous medium.

### RNA Extraction and Quantitative Real-Time PCR (RT-*q*PCR)

For RNA isolation, the peri-implant mucosa and gingiva were separated into submucosa and epithelium layers *via* incubation with Dispase II solution. The different layers were then homogenized in 1 ml TRI reagent (Sigma) using electric homogenizer. After homogenization, the homogenates were centrifuged at 12,000 × *g* for 10 min at 5°C to remove the insoluble material. The clear supernatants were then transferred to fresh tubes and 0.2 ml of chloroform was added to each tube. The tubes were covered tightly, vortexed for 15 s, and left for 10 min at RT. The resulting mixture was centrifuged at 12,000 × *g* for 15 min at 5°C and the colorless upper aqueous phase (containing RNA) was transferred to a fresh tube and 0.5 ml of 2-propanol was added to the tube. The samples stand for 5 min at RT and then centrifuged at 12,000 × *g* for 10 min at 5°C. The RNA precipitate forms a pellet on the side and bottom of the tube. The supernatant was removed and RNA pellet was washed by adding of 1 ml of 75% ethanol per sample, and then centrifuged the samples at 7,500 × *g* for 5 min at 5°C. The RNA pellet was air dried for 5–10 min and an appropriate volume DEPC solution was added. To synthesize cDNA from the RNA pellet, the qScript™ cDNA Synthesis Kit, 95047-100 (Quanta-BioSciences Inc.) was employed. RT-*q*PCR reaction was performed in a 20-µl reaction mixture using Power SYBR Green PCR Master Mix (Quanta-BioSciences Inc.). The following reaction conditions were used: 10 min at 95°C, 40 cycles of 15 s at 95°C and 60 s at 60°C. The samples were normalized to the 18 s as control mRNA, by change in cycling threshold (ΔCT) method and calculated based on 2^−ΔCT^.

### Differentiation Cultures of LC-Like Cells

The femur was isolated, cleaned from soft tissues in RPMI 1640 and soaked in 70% ethanol for 1 min for sterilization. The femur was then washed with sterile PBS and the bone ends was removed by sterile scissors. BM cells eluted from the bone by flushing them several times using sterile syringe filled with RPMI 1640, and the cells were then washed, treated with ACK solution for 3 min on ice, washed again and counted. BM cells (5 × 10^5^ cells/well) in 24-well plates (Nunc) were cultured with complete RPMI media [450 ml RPMI 1640, 50 ml FCS, 5 ml l-glutamine, 50 µM β-mercaptoethanol, penicillin (100 U/ml), streptomycin (100 µg/ml), and gentamicin (50 µg/ml)] supplemented with GM-SCF (100 ng/ml), TGF-β1 (10 ng/ml) for 5 days to induce their differentiation into LC-like cells as previously described (31). To examine the impact of Ti ions on LC, we applied Ti standard solution (1,000 µg/ml Ti in H_2_O, Sigma), at day 0, in various dilutions to the cultures. The cultures were then washed and stained with the noted antibodies for flow cytometry analysis.

### Statistical Analysis

Data were expressed as mean ± SEM. Statistical tests were performed using Student’s *t*-test. *p* < 0.05 was considered significant. *p < 0.05, **p < 0.01, ***p < 0.001.

## Results

### Establishment of Experimental Dental Implant Model in Mice

**Figure 1:**
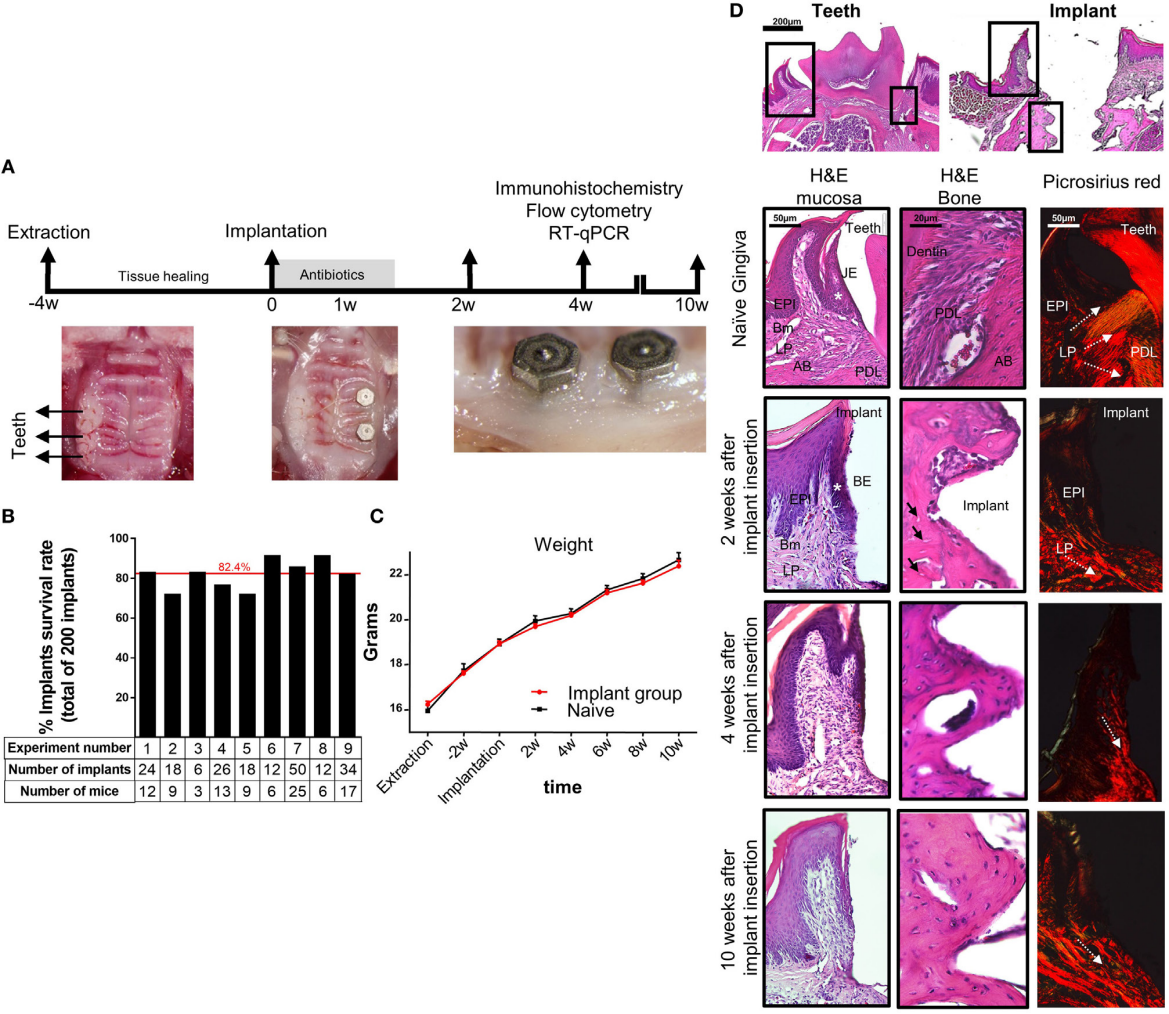
Establishment of a murine model of titanium dental implants. **(A)** Schematic presentation of the experimental setting for insertion of titanium dental implant. Four weeks after tooth extraction two dental implants were inserted in the first and second molar regions of the alveolar bone. The mice were treated for 1 week with antibiotics in the drinking water and soft diet and analyzed 2, 4, and 10 weeks later. **(B)** Bar graph demonstrates the average cumulative survival rate of both implants in nine independent experiments (*n* = 3–25 implanted mice per experiment). The red line indicates the weighted average mean value. **(C)** Mouse weight in implanted and control mice during 10 weeks post implant insertion, presented as the mean ± SEM (*n* = 3–25 mice per group). Representative results of one out of four independent experiments are presented. **(D)** Images of H&E and Picrosirius red staining of the gingiva, peri-implant mucosa, and bone taken at 2, 4, and 10 weeks after implant insertion. The upper images visualize the orientation of the tissue with respect to the location of interest presented as squares. White asterisk marks the junctional epithelium (JE) around teeth and its equivalent the barrier epithelium (BE) around implants. Black arrows indicate empty osteocyte lacunae. White dotted arrows specify the collagen bundle orientation in the tissues. Representative results of one out of three independent experiments are presented. In each experiment, at least three mice per group were examined. Abbreviations: EPI, epithelium; Bm, basement membrane; LP, lamina propria; PDL, periodontal ligament; AB, alveolar bone.

To study basic immunological and microbial mechanisms involved in the placement of dental implants, a murine model is highly desirable since vast experimental tools and transgenic mice are available. We thus established a novel approach to orally place two titanium dental implants in mice (Figure 1A). In this model, the left upper molars were extracted and the mice left to heal for 4 weeks. A custom made titanium implants (MIS Implants Technologies, Israel) were then placed into two sites correlating with the first and second molar sites. As depicted in Figure 1B, the total average survival rate of 200 implants located at the posterior maxilla was over 80%. This rate is in accordance with the survival rate reported in human studies, emphasizing the efficacy and strength of our experimental model (32, 33). The implanted mice had no significant clinical signs and gained weight in a similar kinetics as the control sex- and aged-matched naïve mice (Figure 1C), indicating the mice were in a good health after the implantation. Time course histological analysis of the peri-implant region detected a newly formed bone 2 weeks post-implantation with high number of empty osteocytic lacunas that decreased over time (Figure 1D). We next used Picrosirius red staining to evaluate the collagen network in the peri-implant connective tissue. As opposed to the gingiva where the collagen fibers were distributed in a fan-shaped pattern forming a tight seal around teeth, the collagen bundles in the peri-implant tissue were located in parallel to the implant, as previously demonstrated for dental implants in human studies (34) with no remarkable changes later on (Figure 1D). Taken together, the described model results in a successful integration of the dental implants and similar histological characteristics as dental implants in humans, thus can be used for further studying how the implants impact local immunity.

### Elevated LC Precursors but Reduced Levels of Fully Developed LCs in the Peri-Implant Epithelium

**Figure 2:**
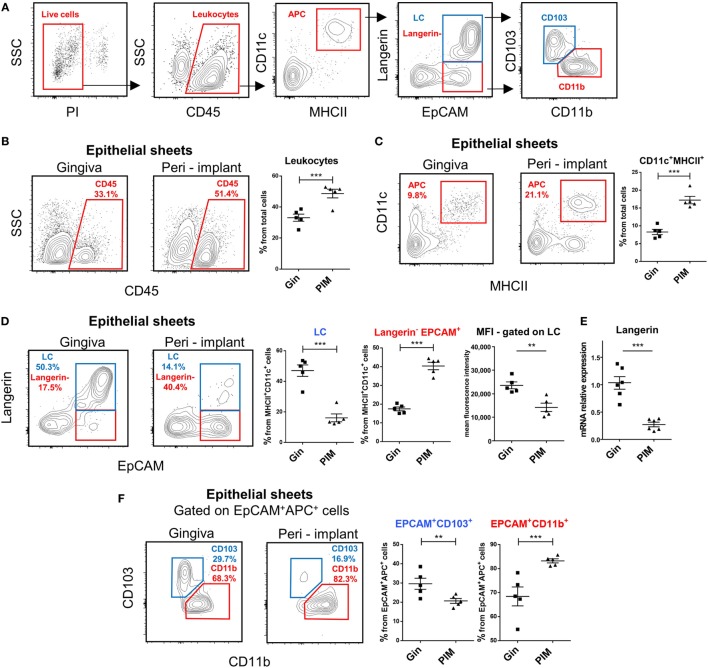
Decreased frequencies of terminally differentiated Langerhans cells (LCs) in the peri-implant epithelium. **(A)** Gating strategy to identify LCs and their precursors in the tissue using flow cytometry. **(B,C)** Frequencies of CD45^+^ leukocytes **(B)** and CD11c^+^MHCII^+^ LC precursors **(C)** in the peri-implant and gingival epithelium 4 weeks after implant insertion. Representative FACS plots and graphs indicates percentages of CD45^+^ and CD11c^+^MHCII^+^ cells from total cells, presented as the mean ± SEM (*n* = 5 per group, each *n* represents oral tissues pooled from three individual mice). Data are representative of one out of three independent experiments. **(D)** Expression of langerin and EpCAM on CD11c^+^MHCII^+^ cells in the peri-implant and gingival epithelium 4 weeks after implant insertion. Representative facs plots and graphs depicting the percentages of EpCAM^+^langerin^+^ (LCs) and EpCAM^+^langerin^neg^ cells form total CD11c^+^MHCII^+^ cells are presented as the mean ± SEM (*n* = 5 per group). Representative graph illustrate the mean fluorescence intensity (MFI) of langerin expression pre-gated on LCs in peri-implant and gingival epithelium presented as the mean ± SEM (*n* = 5 per group). **(E)** Quantification of *langerin* mRNA in epithelial tissue prepared from peri-implant and gingival tissues using quantitative real-time PCR. Graph presents the fold change in gene expression normalized to non-implanted mice and presented as the mean ± SEM (*n* = 6 mice per group). Data are representative of one out of two independent experiments. **(F)** Expression of CD11b and CD103 in epithelial CD11c^+^MHCII^+^EpCAM^+^ cells 4 weeks after implant insertion. Representative FACS plots and graphs illustrating the percentages of the noted subsets presented as the mean ± SEM (*n* = 5 per group). **p < 0.01, ***p < 0.001.

Since previous studies reported opposing results regarding the frequencies of LCs in the peri-implant epithelium, we addressed this issue in our murine system. Epithelial tissues of the peri-implant gingiva were collected 4 weeks after implantation and subjected to flow cytometry analysis (Figure S1 in Supplementary Material). Gingival epithelial tissues from sex- and age-matched naïve mice were used as a control. To identify LCs, the processed epithelial cells were stained with antibodies against CD45, CD11c, MHCII, EpCAM, and langerin, and analyzed based on the gating strategy described in Figure 2A. In some experiments, additional staining with CD11b and CD103 antibodies was performed to further separate the LCs to CD103^+^CD11b^low^ (CD103^+^LCs) and CD11b^+^CD103^neg^ (CD11b^+^LCs) subsets as previously reported (23). As demonstrated in Figure 2B, elevated percentages of CD45^+^ leukocytes were detected in the peri-implant epithelium compared to naïve gingiva. Moreover, the frequencies of LC precursors, identified as CD45^+^CD11c^+^MHCII^+^ cells, were also significantly increased in the tissue (Figure 2C). Nevertheless, despite the increment of their precursors, the frequencies of fully developed LCs in the peri-implant epithelium were considerably reduced (twofold to threefold reduction) in comparison to the control group (Figure 2D). Further examination revealed that a large fraction of the CD45^+^CD11c^+^MHCII^+^ cells expressed EpCAM but not langerin, and even the mean fluorescence intensity of langerin on the remaining LCs in the peri-implant was lower than control gingival LCs. The reduction in langerin expression was also confirmed using RT-*q*PCR (Figure 2E). Importantly, the LC population in the contralateral intact gingiva of the implanted mice was not altered, indicating the confined effect of the implants (Figure S2 in Supplementary Material). We then examined which LC subset is mostly affected by the implant. Since LC numbers in the peri-implant mucosa were too low for such analysis, we gated on CD45^+^CD11c^+^MHCII^+^EpCAM^+^ cells (i.e., the direct precursors of LCs), and found that CD103^+^ LCs were mostly affected by the implant (Figure 2F).

**Figure 3:**
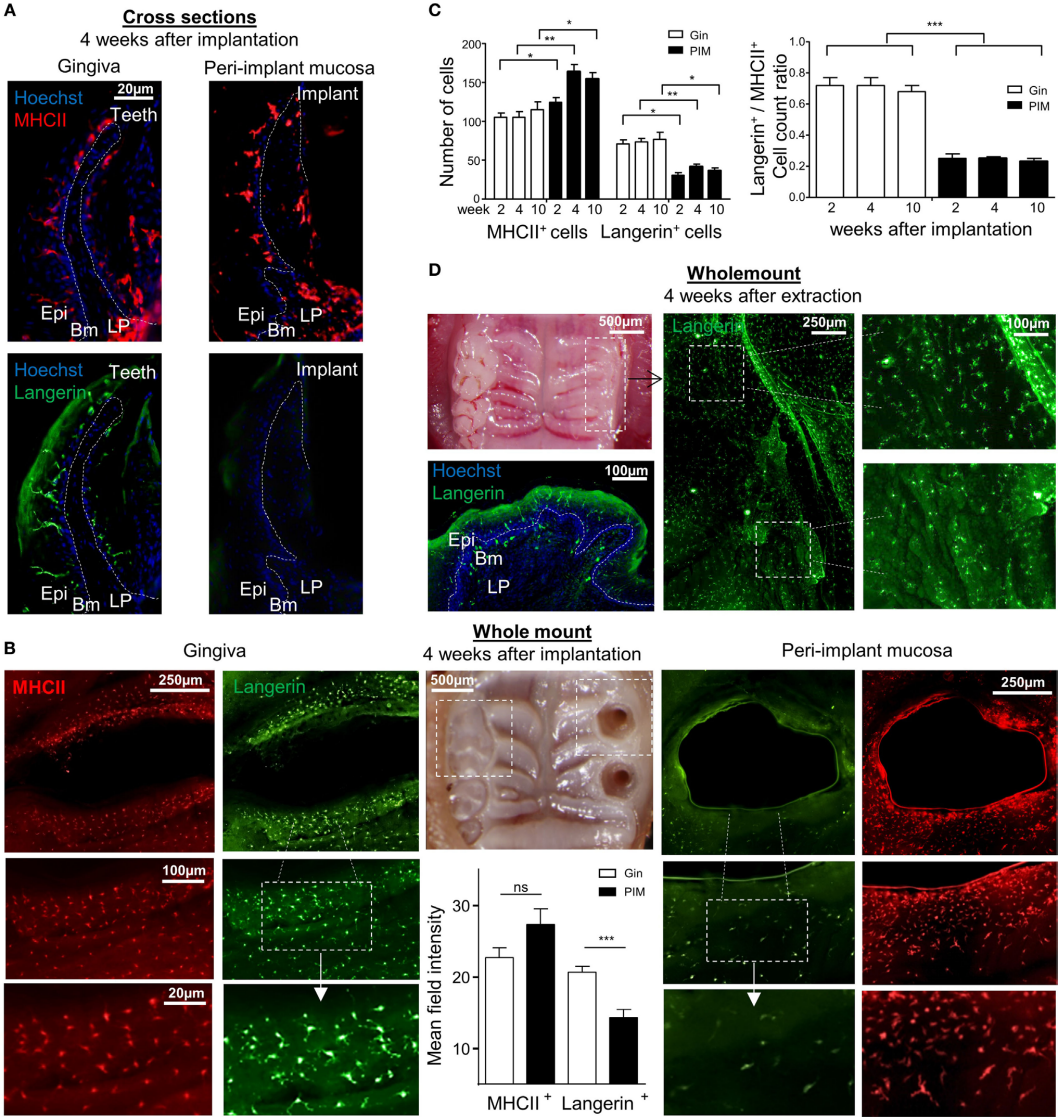
Visualization of langerin and MHCII expression in the peri-implant mucosa. **(A)** Immunofluorescence imaging of the peri-implant and gingival cross sections tissues prepared 4 weeks after implantation stained against MHCII (red), langerin (green), and hoechst (blue) for nuclear visualization. Representative immunofluorescence images of two independent experiments are shown. In each experiment, at least three mice were examined. **(B)** Epithelial layers prepared from peri-implant and gingival tissues 4 weeks after implant insertion, stained for (red), langerin (green), and hoechst (blue). Whole mount immunofluorescence images from one out of two independent experiments are shown, at least three mice were examined in each experiment. Bar graphs represent the mean field staining intensity of langerin and MHCII in the epithelium, shown as the mean ± SEM. The average pixel intensity was calculated for each tissue using ImageJ software by 100 µm circumferential countering the epithelium around implant and teeth (*n* = 3 mice per group). Representative images of one out of two independent experiments are shown. **(C)** Graphs presents the total numbers of MHCII-positive or langerin-positive cells per a field of view, as well as the relative ratio of the noted cells in epithelial sheets taken 2, 4, and 10 weeks after implant insertion, presented as the mean ± SEM (*n* = 6 mice per time points). Data of one out of two independent experiments are presented. **(D)** Four weeks after tooth extraction, epithelial tissues were removed from the alveolar process and subjected to immunofluorescence staining as described above. Representative images of one out of two independent experiments are provided, three mice were analyzed in each experiment. *p < 0.05, **p < 0.01, ***p < 0.001.

To further visualize the LCs in the peri-implant epithelium, an immunofluorescence staining on gingival histological cross sections and on whole epithelial layers was executed. Four weeks after implantation, the tissues were prepared from individual mice and stained with antibodies against MHCII and langerin. Concurring with the flow cytometry data, MHCII-positive cells with a morphology of DCs were clearly visualized in the epithelium of both the peri-implant and naïve gingiva epithelium (Figures 3A,B). The number of MHCII^+^ cells was also significantly higher in the peri-implant epithelium in comparison to normal gingiva, and this trend last up at least to 10 weeks post-implantation (Figure 3C). On a contrary, langerin-positive cells were very scarce in the peri-implant epithelium, and langerin staining intensity of the residual LCs in this region was much weaker in comparison to control samples (Figures 3A,B). The reduction of langerin-positive cells was also detected for at least up to 10 weeks post-implantation (Figure 3C). To verify that the lack of langerin-positive cells was not resulting from the surgical procedure performed prior to implant placement, we stained epithelial tissue 4 weeks after tooth extraction just before the implantation. As shown in Figure 3D, langerin-positive cells were easily visualized in the epithelium, indicating that langerin-positive cells were normally present in the epithelium prior to the implantation. Collectively, these results suggest that terminal maturation of LCs is impaired in the peri-implant epithelium. The accumulation of CD45^+^CD11c^+^MHCII^+^EpCAM^+^ LC precursors, representing a transient stage in the development of oral mucosal LCs, proposes that LC differentiation might be dysregulated in the peri-implant epithelium.

### Cytokines and Chemokines Mediating the Development of Oral LCs Are Differentially Dysregulated in the Peri-Implant Mucosa

**Figure 4:**
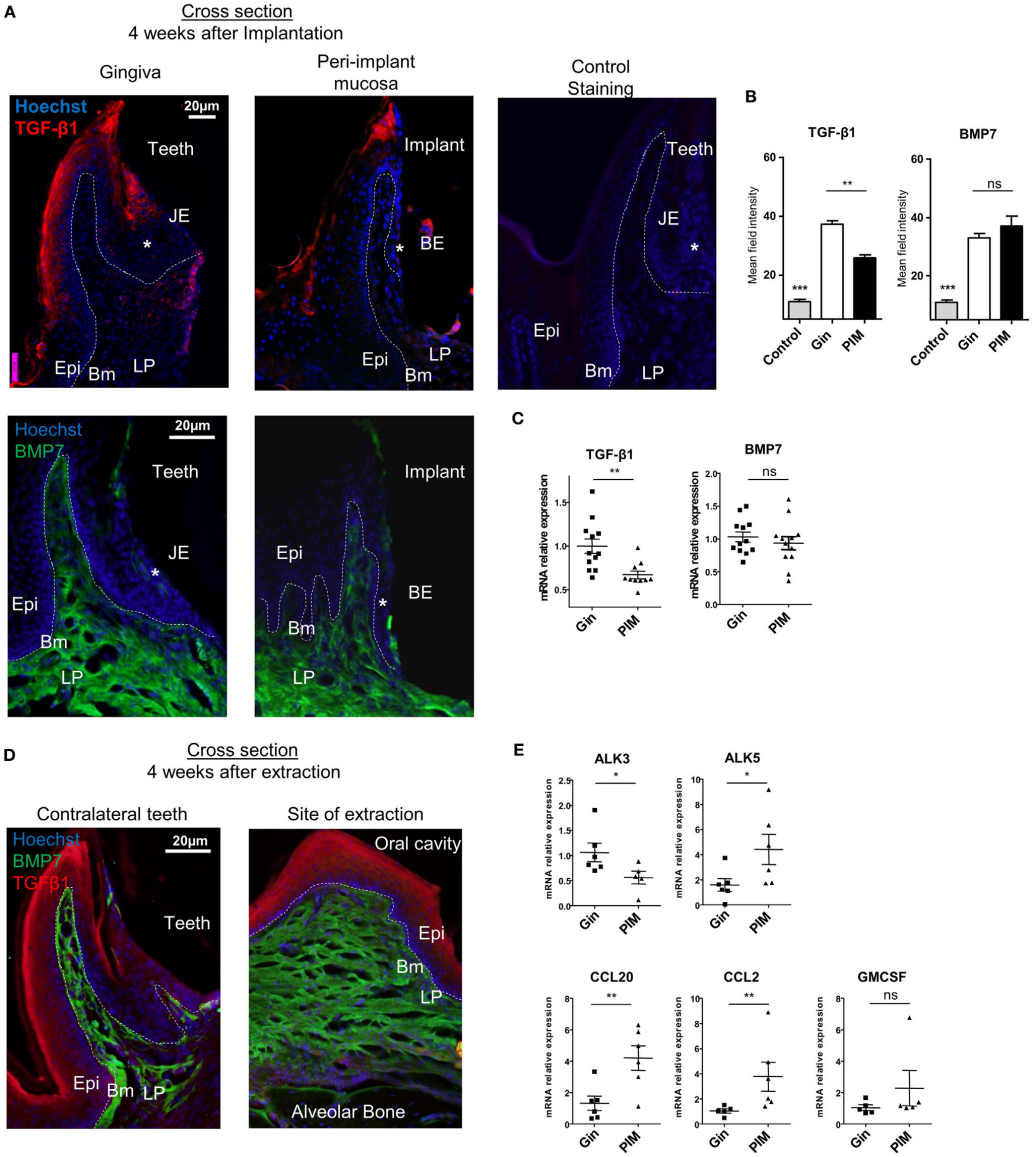
Dysregulated expression of molecules mediating Langerhans cell differentiation in the peri-implant mucosa. **(A)** Immunofluorescence cross sections of peri-implant and gingival tissues stained 4 weeks after implantation against TGF-β1 (red), bone morphogenetic protein 7 (BMP7) (green), and hoechst (blue). Negative control represents staining with the secondary antibody only. Representative immunofluorescence images of two independent experiments are shown. In each experiment, at least three mice were examined. **(B)** Bar graphs represent the mean field staining intensity of TGF-β1 and BMP7 in the epithelium and submucosa, respectively, shown as the mean ± SEM. The average pixel intensity was calculated for each slide using ImageJ software by countering the epithelium or the submucosa from nine different slides for each group (*n* = 3 mice per group). Representative images of two independent experiments are shown. **(C)** Four weeks after implant insertion, TGF-β1 and BMP7 mRNA expressions were quantified in the epithelium and submucosa, respectively, by RT-*q*PCR. Graph presents the fold change in gene expression normalized to non-implanted mice and presented as the mean ± SEM (at least 10 mice per group were analyzed). Data are representative of two pooled independent experiments. **(D)** Four weeks after teeth extraction, immunofluorescence cross sections of the alveolar process at extraction sites were stained as described in **(A)**. Representative images of one out of two independent experiments are provided, three mice were analyzed in each experiment. **(E)** Graph presents the fold change in gene expression of activing-like kinase 3 (ALK3), ALK5, CCL20, CCL2, and GM-CSF in the epithelium, normalized to non-implanted mice 4 weeks after implantation, presented as the mean ± SEM (*n* = 6 mice per group). Data are representative of one out of two independent experiments. *p < 0.05, **p < 0.01.

As the aforementioned results suggest that the differentiation of LCs in the peri-implant mucosa is alerted, we next examined the expression of molecules which are known to mediate this process. Expression of TGF-β1 and BMP7, the predominant cytokines instructing LC differentiation, was first analyzed using immunofluorescences staining on gingival cross sections. TGF-β1 expression was detected in the suprabasal epithelia layers of the oral epithelium and also in the sulcular/junctional epithelium (JE) facing the tooth surface (Figure 4A). In the peri-implant mucosa, however, expression of TGF-β1 was reduced in the oral epithelium and nearly absent in the barrier epithelium facing the implant (the equivalent of the JE in normal gingiva). Quantification of TGF-β1 staining intensity in several fields of view (Figure 4B), or the levels of *Tgf-*β1 mRNA by RT-*q*PCR (Figure 4C), further confirmed the reduction in the expression of this cytokine in the epithelium. In contrast to TGF-β1, BMP7 expression which is restricted to the LP (24) was not affected in the peri-implant mucosa (Figures 4A–C). Of note, the reduction in TGF-β1 expression was not due to the tooth extraction procedure, since TGF-β1 was comparably expressed in the epithelium of both the tooth extraction site and the contralateral intact gingiva (Figure 4D). As TGF-β1 and BMP7 were proposed to impact LC development *via* both ALK5 and ALK3 receptors or ALK3 only, respectively (24, 35), the expression of these receptors was also quantified using RT-*q*PCR. As depicted in Figure 4E, whereas the mRNA levels of *Alk5* were upregulated in the peri-implant epithelium compared to normal gingiva epithelium, the levels of *Alk3* mRNA were significantly reduced. Next, mRNA expression of the chemokines *Ccl2* and *Ccl20* was quantified in the epithelium, since we previously reported that these chemokines are differentially expressed in the epithelium and mediate LC recruitment (24, 35). Expression of both cytokines was upregulated in the peri-implant epithelium compared to the control group (Figure 4E). Expression of *Gm-csf*, an additional cytokine required for APCs and LCs differentiation, was not modified in the peri-implant epithelium (Figure 4E). These results thus suggest that TGF-β1/ALK5 as well as TGF-β1/ALK3 signaling are dysregulated in peri-implant mucosa. As these singling pathways mediate the differentiation rather than recruitment of LC precursors to the epithelium (24), their dysregulation correlates well with the presence of CD45^+^CD11c^+^MHCII^+^EpCAM^+^ LC precursors in the epithelium.

### Titanium Ions Inhibit the Expression of Langerin on *In Vitro* Generated LC-Like Cells

**Figure 5:**
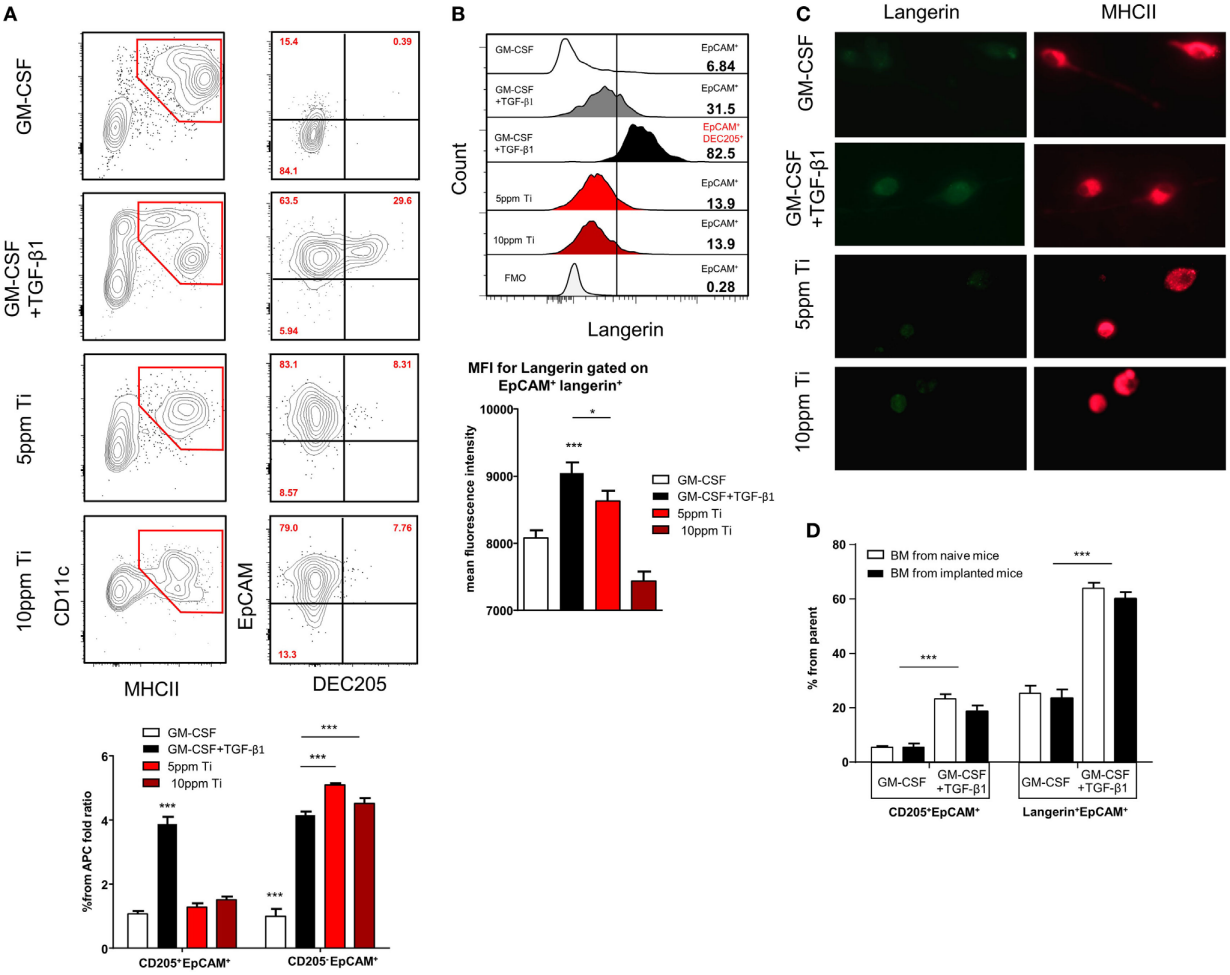
Titanium ions altered *in vitro* differentiation of Langerhans cell (LC)-like cells from BM precursors. **(A)** Frequencies of CD11c^+^MHCII^+^ cells and LC-like cells (CD11c^+^MHCII^+^EpCAM^+^DEC205^+^) 5 days after incubation with GM-CSF or TGF-β1 + GM-CSF in the presence or absence of 5 and 10 ppm of Ti ions. Representative FACS plots and graphs indicates the fold change in the percentage of EpCAM^+^DEC205^+^ (LC-like cells) and EpCAM^+^DEC205^neg^ cells from total CD11c^+^MHCII^+^ population (percentages were normalized to the GM-CSF group) presented as the mean ± SEM (*n* = 6 mice per group). Data are representative of one out of four independent experiments. **(B)** Representative histogram plots illustrate the frequencies of langerin expression on pre-gated EpCAM^+^ and EpCAM^+^DEC205^+^ cells after 5 days of culture. Bar graph specifies the mean fluorescence intensity (MFI) of langerin expression on pre-gated cells shown as the mean ± SEM (*n* = 6 mice per group). Data are representative of one out of two independent experiments. **(C)** Cells were collected from the LC differentiation cultures after 5 days and stained against langerin (green) and MHCII (red). Representative immunofluorescence images presenting the expression of langerin and MHCII in each culture condition. Data of one out two independent experiments are shown. **(D)** BM cells purified from implanted and non-implanted mice were cultured with GM-CSF or GM-CSF + TGF-β1 and 5 days later were analyzed by flow cytometry. Bar graph presents the frequencies of LCs-like cells based on the expression of EpCAM, DEC205 an langerin from the CD11c^+^MHCII^+^ cells representing the mean ± SEM (*n* = 3 mice per group). Data are representative of one out of two independent experiments *p < 0.05, ***p < 0.001.

It has been demonstrated that titanium ions are released from titanium implants (9, 13) and such ions are capable of altering DC function *in vitro* (11, 36). We thus asked whether titanium ions might also modify the differentiation of LC from BM cells, the direct precursors of oral mucosal LCs (23). To induce LC differentiation, BM cells were cultured for 5 days with serum-containing media supplemented with GM-CSF and TGF-β1. Titanium ions were also added to the cultures to examine their capacity to inhibit or modulate LC differentiation. First, we examined the toxicity of various concentrations of titanium ions by quantifying cellular viability in the cultures (Figure S3A in Supplementary Material). Among the various concentrations tested, 5 and 10 ppm of titanium ions resulted in high percentages of live cells >95 and 60%, respectively. In addition, the pH value of the serum-containing differentiation cultures was not affected by the presence of any titanium ions concentration tested (Figure S3B in Supplementary Material). Next, we quantified the generation of LCs by gating on EpCAM^+^DEC205^+^ cells among the CD11c^+^MHCII^+^ population, as these were previously proven to represent LC-like cells (37). Cultures containing GM-CSF + TGF-β1 were able to induce a large fraction of EpCAM^+^DEC205^+^ LC-like cells (Figure 5A). Moreover, an additional subset of the CD11c^+^MHCII^+^ cells acquired a phenotype of EpCAM^+^DEC205^neg^ cells, a population with a potential to differentiate to LC-like cells (37). The addition of titanium ions to the cultures failed to generate EpCAM^+^DEC205^+^ LC-like cells and only the EpCAM^+^DEC205^neg^ population was detected in these cultures. Further characterization revealed that only LC-like cells generated in the absence of titanium ions were able to upregulate langerin expression (Figure 5B; Figure S4 in Supplementary Material). The impact of titanium ions on langerin expression was also verified using immunofluorescences analysis by staining the cells with antibody against MHCII and langerin. As depicted in Figure 5C, expression of langerin, but not MHCII, on cells derived from titanium ions-containing cultures was greatly reduced compared to control group. To examine whether titanium ions are capable also to alter the activation of the MHCII^+^CD11c^+^EpCAM^+^ cells, we employed a different culturing strategy. BM cells were cultured for 3 days with GM-CSF and TGF-β1 and then exposed to titanium ions for 2 days. Higher expression of the co-stimulatory molecules CD86 and CD40 was detected on the surface of MHCII^+^CD11c^+^EpCAM^+^ cells, suggesting the titanium ions induce the maturation of these APCs (Figure S5 in Supplementary Material). Next, since titanium ions dysregulates the capacity of BM cells to evolve into LC-like cells, we asked whether titanium implants are capable to alter the differentiation capacity of BM cells systemically rather than locally. For this we isolated BM cells from implanted and naïve mice and cultured them with GM-CSF + TGF-β1. Analysis of the cultures indicated that BM cells from both groups of mice have comparable differentiation capability to LC-like cells (Figure 5D). Taken together, these results suggest that titanium ions dysregulate the development of LC-like cells *in vitro*, and preferentially induce cells that are not fully differentiated into LCs but are rather more activated. Moreover, this regulatory function appears to be a local effect rather than systemic, impacting the development of LCs only in areas adjacent to the implant.

### High Leukocyte Content in the Peri-Implant Mucosa With No Evidence of an Acute Inflammation

**Figure 6:**
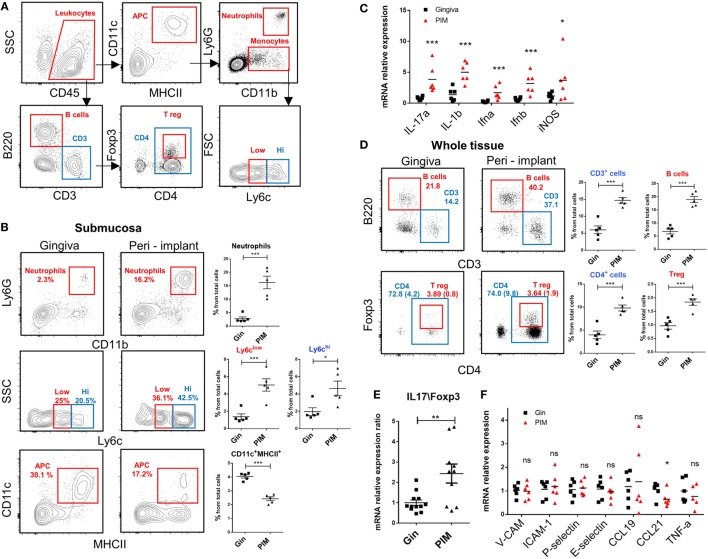
An increased leukocyte infiltrate in the peri-implant mucosa. **(A)** Gating strategy to identify by flow cytometry the noted leukocyte subsets in the tissue. **(B)** Flow cytometry plots illustrate the incidence of neutrophils (Ly6G^+^CD11b^+^), monocytes (CD11b^+^Ly6C^high\low^), antigen-presenting cells CD11c^+^MHCII^+^ in the submucosa of the peri-implant and gingiva 4 weeks after implant insertion. Correlating graphs demonstrates the frequencies of the noted immune subsets from the total cell count, presented as the mean ± SEM (*n* = 5 per group, each *n* represents tissues pooled from three individual mice). **(C)** mRNA expression levels of proinflammatory cytokines were calculated in the peri-implant epithelium 4 weeks after implant insertion, presented as the mean ± SEM (*n* = 6 per group, each *n* represents tissues pooled from three individual mice). **(D)** Frequencies of total lymphocytes (CD3^+^), B cells (B220^+^CD3^−^), T helper cells (CD3^+^CD4^+^), and T regulatory (Treg) cells (CD3^+^CD4^+^FOXP3^+^) from the whole peri-implant and gingiva tissues are presented in flow cytometry plots. Frequencies of CD4^+^ T cells and Treg cells from total cells are provided in brackets within the flow cytometry plots. Graphs indicate the frequency of each cells subset from the total cell population, presented as the mean ± SEM (*n* = 5 per group, each *n* represents tissues pooled from three individual mice). Data are representative of one out of two independent experiments. **(E,F)** Four weeks after implant insertion, the mRNA levels of the noted genes were quantified in the peri-implant and gingiva lamina propria using RT-*q*PCR. **(E)** Ratio of IL-17 and Foxp3 expression level. Data are representative of two independent experiments. **(F)** Graph presents the fold change in gene expression normalized to non-implanted mice and represent the mean ± SEM (*n* = at least five mice per group). Data are representative of one out of two independent experiments. *p < 0.05, **p < 0.01, ***p < 0.001.

We next analyzed the various leukocytes present in the peri-implant mucosa 4 weeks after implantation. Figure 6A demonstrates the gating strategy employed to identify innate and adaptive leukocytes in the peri-implant submucosa and normal gingiva as a control. Higher levels of neutrophils, Ly6C^high^ and Ly6C^low^ monocytes were found in the peri-implant tissue compared to naïve gingiva (Figure 6B). Interestingly, the frequencies of CD11c^+^MHCII^+^ APCs in the peri-implant submucosa were significantly lower than those detected in the submucosa of normal gingiva. In correlation with the increased innate cell infiltrate in implanted mice, we also found an elevated expression of *Il-17a, Il-1b, Ifna, Ifnb*, and *Nos2* (iNOS) in the peri-implant mucosa (Figure 6C). With regards to adaptive lymphocytes, the percentages of B and T cells were also elevated in the peri-implant mucosa, particularity the CD4^+^ T cell subset. A larger number of these CD4^+^ T cells in the peri-implant mucosa was stained positively to FOXP3, indicating that compared to normal gingiva this tissue contains more Treg cells (Figure 6D). Since mucosal homeostasis can be reflected by the ratio of Th17 and Treg cells, we next calculated the relative expression levels of *Il-17a* versus *Foxp3* in the tissue. As shown in Figure 6E, the *Il-17A*/*Foxp3* ratio was higher in the peri-implant mucosa in comparison to normal gingiva, indicating that the implant modified tissue homeostasis by increasing inflammatory milieu. We then asked whether such immunological state, recorded 4 weeks after implant placement, represents an acute inflammation in the peri-implant mucosa. To address this question we first quantified the expression of the proinflammatory cytokine *Tnf-a* and found comparable levels in the peri-implant mucosa and normal gingiva (Figure 6F). Moreover, expression levels of *P-selectin, E-selectin, Icam-1*, and *Vcam-1*, adhesion molecules that are upregulated on activated endothelium, were similar in both tissues (Figure 6F). We also examined the expression of *Ccl21* and *Ccl19*, chemokines mediating migration of DCs to the draining LNs and found reduced levels of *Ccl21* around implants but not *Ccl19* (Figure 6F). These results suggest that 4 weeks after implant placement, the peri-implant mucosa contains an elevated leukocyte content, whereas there is no indication to the presence of acute inflammation in the tissue at this time point. With regards to the CD11c^+^MHCII^+^ APCs, it is possible that their reduced frequency in the peri-implant submucosa is due to increased translocation to the epithelium rather then accelerated migration to the LNs.

## Discussion

Using a murine model of titanium dental implants, this study shows the absence of fully differentiated LCs in the epithelium of the peri-implant mucosa. We provided both *in vivo* and *in vitro* evidence demonstrating that titanium implants and titanium ions, respectively, are capable of impairing local LC development. As a result, oral LCs expressed EpCAM but failed to upregulate langerin expression, suggesting a partial differentiation of these cells. Previous human studies employing various markers to identify human LCs such as MHCII, CD1a or S-100, reported contradicting results regarding the frequencies of LCs in the peri-implant mucosa (26–28, 30). While the expression kinetics of these markers on human LCs are not clear yet, they are likely to be expressed at different differentiation stages. Thus, it is expected that analysis based on only a single or two markers will provide diverse results.

It has been shown that the first differentiation step of oral LCs is mediated by BMP7/ALK3 signaling, which upregulate expression of E-cadherin, CCR2, and CCR20, enabling the translocation of LC precursors to the epithelium (24). The accumulation of CD11c^+^MHCII^+^EpCAM^+^ cells in the peri-implant epithelium suggests that this early differentiation step is intact or even accelerated in implanted mice. In line with this notion, BMP7 expression was not altered in the submucosa, whereas epithelial expression of CCL2 and CCL20 was upregulated. Therefore, titanium implants or titanium ions are considered to impact mainly the second differentiation step of oral LCs that is driven by TGF-β1 in the epithelium. Indeed, expression of TGF-β1 was significantly reduced in the epithelium of the peri-implant mucosa. It is currently unclear how titanium implants or ions regulate TGF-β1 expression, nevertheless, there are indications in human implants and *in vitro* cultures that titanium can modulate TGF-β1 expression (38, 39). Besides TGF-β1, titanium implants were also capable to upregulate expression of its canonical ALK5 receptor while reducing the non-canonical ALK3 receptor. Signaling *via* TGF-β1 and ALK5 was shown to be important for LC homeostasis as it prevents their spontaneous activation and migration to the LNs (40, 41). Concurring with this notion, the upregulation of ALK5 might compensate for the reduced TGF-β1 levels, resulting in the accumulation of the partly developed LCs in the peri-implant epithelium. Yet, TGF-β1/ALK5 signaling is also crucial for efficient differentiation of oral LCs, since differentiation of these cells was shown to be inhibited but not absent in mice lacking ALK5 in CD11c^+^ cells (24). Such ALK5-independent development of oral LCs is likely to be mediated by ALK3 as previously suggested (35). Nevertheless, since ALK3 expression was reduced in the peri-implant epithelium, TGF-β1/ALK5 signaling seems to play the major role in the partial development of the oral LCs. Interestingly, we recently proposed that TGF-β1/ALK3 signaling in the epithelium can mediate langerin expression on oral LCs (24). Accordingly, the reduced expression of both ALK3 and TGF-β1 in the peri-implant epithelium, might explain the lack of langerin expression by local LCs.

In comparison to normal gingiva around teeth, the peri-implant mucosa contains larger infiltrate of innate and adaptive leukocytes 4 weeks after implantation, with no indication to the existence of acute inflammation. An inflammatory infiltrate in the peri-implant mucosa was reported also in clinically healthy implants 6 months after implant insertion (42), which support our findings and also emphasizing the relevance of our model to the clinical situation. Nonetheless, such a phenomenon could simply represent a transitional immunological state of the peri-implant mucosa, which might eventually return to steady state level as in normal gingiva. The gradual reduction in the size of the peri-implant infiltrate that was reported in humans supports this assumption (43). However, it is not clear whether the peri-implant mucosa will indeed reach a “normal” steady state as in the gingiva. This highlights an alternative possibility for the development of an alerted steady state conditions in the peri-implant mucosa. This hypothesis may explain the increased susceptibility of the implant to infection, as based on the Th17/Treg balance, the immunological steady state of the peri-implant is more “inflamed” than normal gingiva. Such condition might eventually lead to a microbial dysbiosis, involving the accumulation of inflammophilic bacteria that will induce disease as we previously reported (44).

The extent of the dysregulation of LC development affects the immune response of the peri-implant mucosa is still unknown. LCs were shown to regulate immunological and microbial homeostasis of the oral mucosa (24), and to play a protective role during infection with an oral pathogen (25). In both cases, LC function was mediated, in part, by their capacity to induce Treg cells that resolve gingival inflammation. Since the Th17/Treg balance is augmented in the peri-implant mucosa compared to normal gingiva, it is likely that the partially differentiated LCs might have reduced capacity to generate Treg cells. The specific decrease in CD103^+^ LCs, a subset that is greatly affected by the microbiota (24), further suggest that the reduction in local LCs might affect the tissue. Nevertheless, further investigation will be required to assess this issue directly.

In summary, the present study provides novel insights into the impact of titanium implant on oral LCs, the major APC subset residing in the epithelium and regulating oral mucosal homeostasis. Besides increasing our understanding on immunological dysfunction associated with insertion of titanium dental implants, this study highlights the sequential differentiation process of oral mucosal LCs. Moreover, it suggests that environmental factors have the capacity to interfere with LC development and subsequently alter oral mucosal homeostasis.

## Ethics Statement

This study was carried out in accordance with the recommendations of “The guide for care and use of laboratory animals,” Hebrew University Institutional Animal Care and Ethics Committee. The protocol was approved by the Hebrew University Institutional Animal Care and Ethics Committee.

## Author Contributions

OH, A-HH, LS, and AW designed the research; OH analyzed the data; OH, NK, GM, TC, SW, MN, and YT performed experiments; A-HH, AW, LS, and OH wrote the manuscript; funding acquisition: AW and A-HH.

## Conflict of Interest Statement

The submitted work was carried out without the presence of any personal, professional, or financial relationships that could potentially be construed as a conflict of interest. The reviewer CB and handling Editor declared their shared affiliation.
