# Supplementary figures and images for: Impaired Differentiation of Langerhans Cells in the Murine Oral Epithelium Adjacent to Titanium Dental Implants
Source: Front Immunol. 2018 Aug 15;9:1712. doi: 10.3389/fimmu.2018.01712 (PMC6103475; doi:10.3389/fimmu.2018.01712)

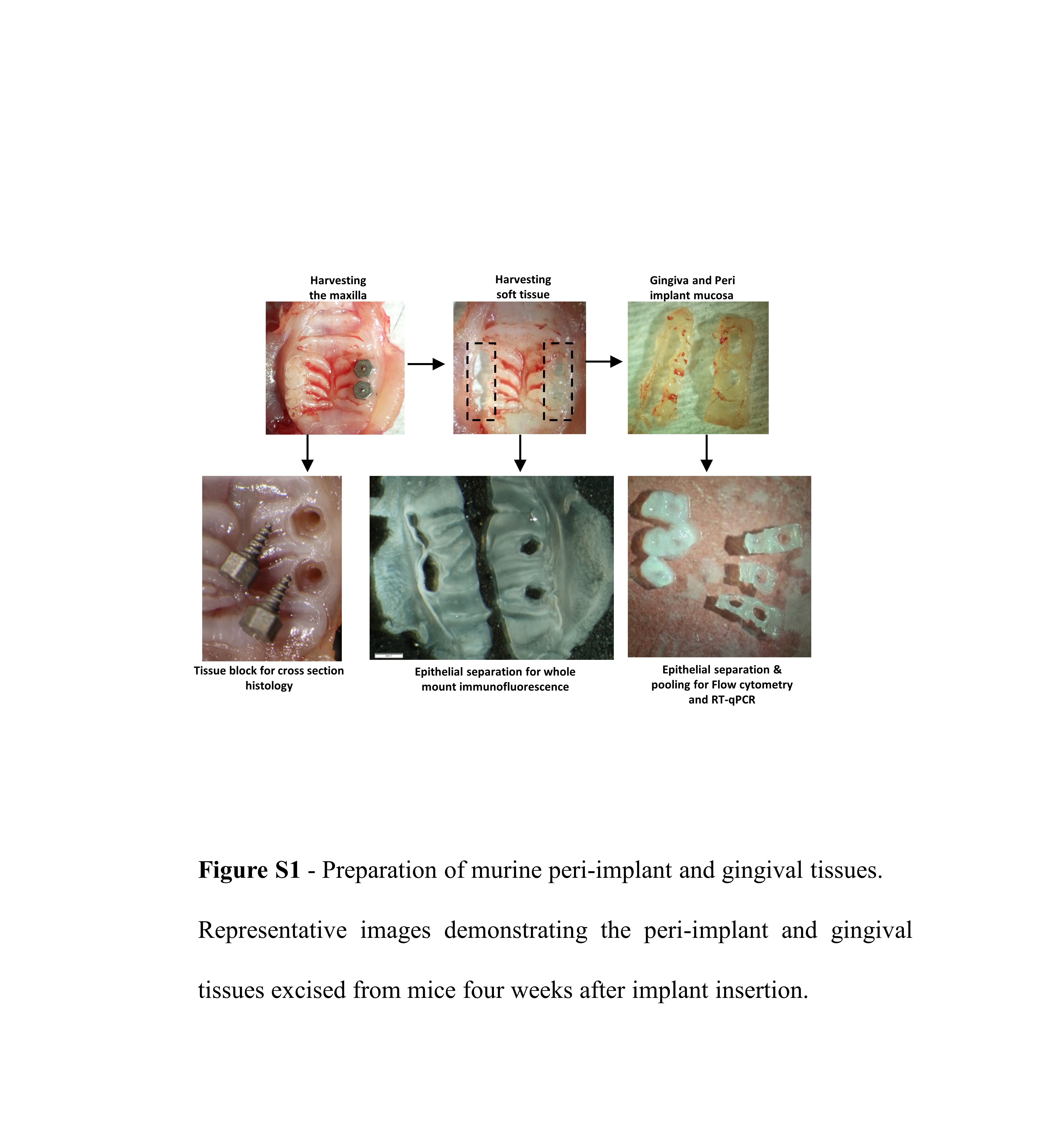

Supplement: Supplementary file 1 [file image_1.jpeg]

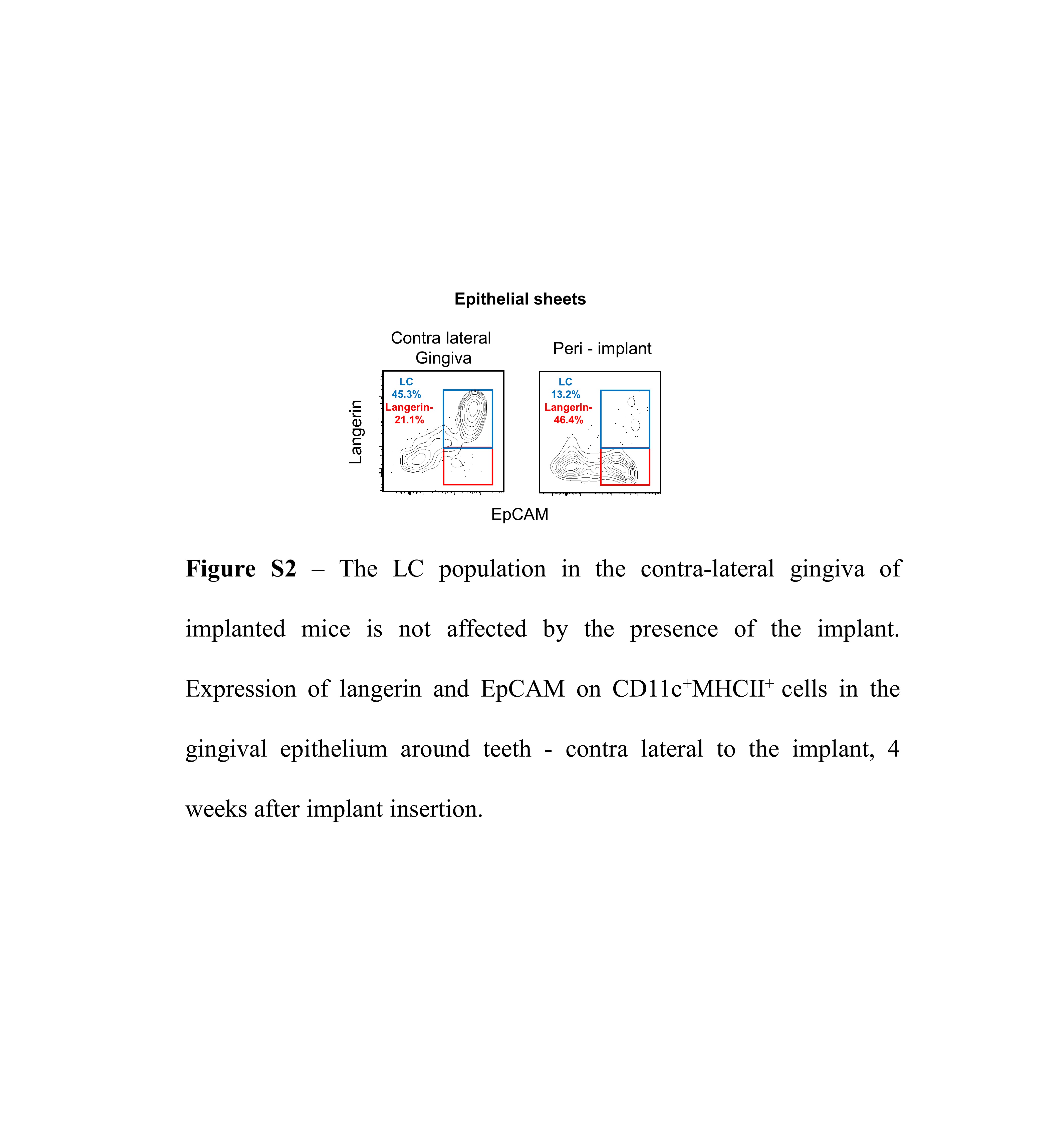

Supplement: Supplementary file 2 [file image_2.jpeg]

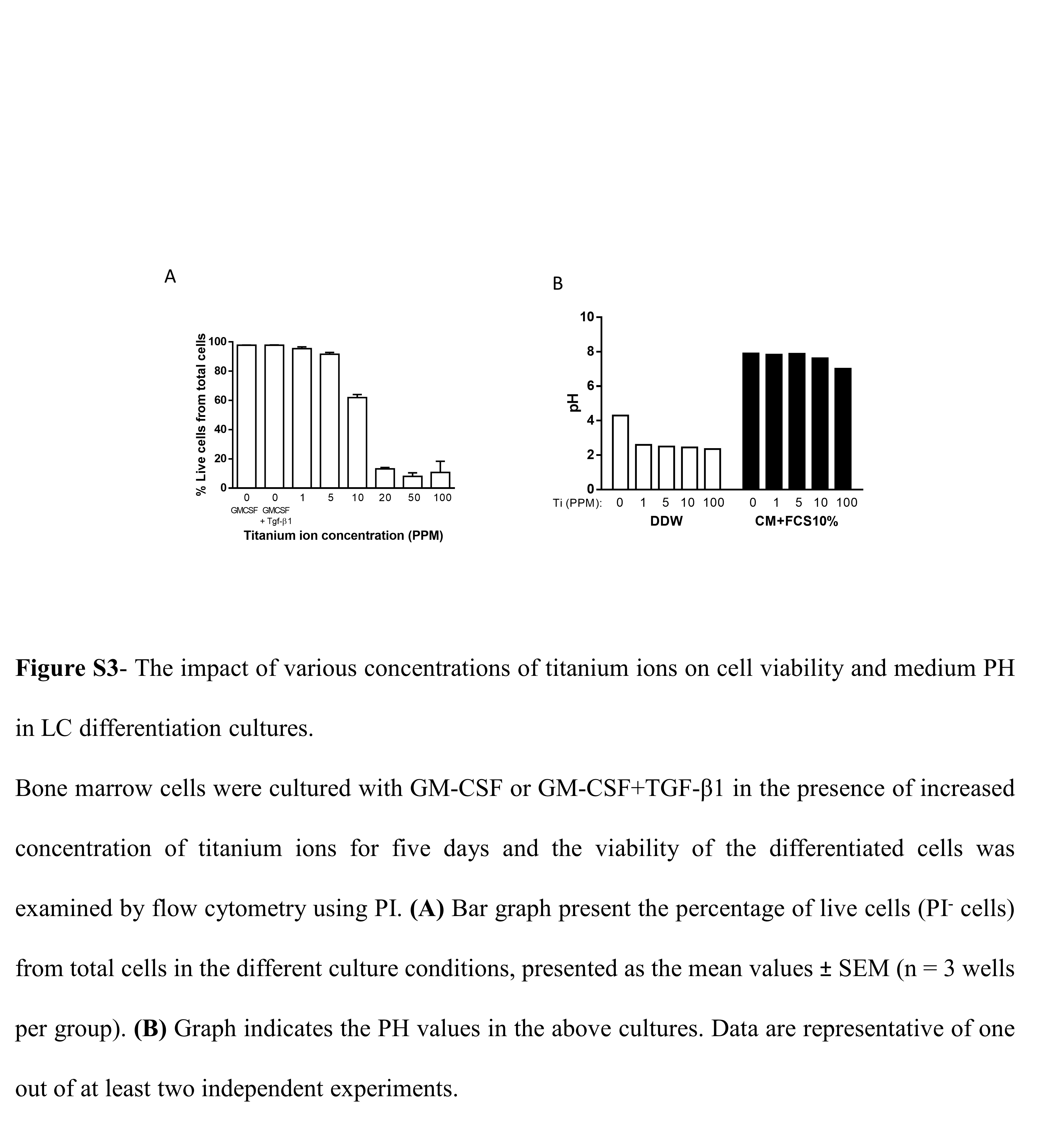

Supplement: Supplementary file 3 [file image_3.jpeg]

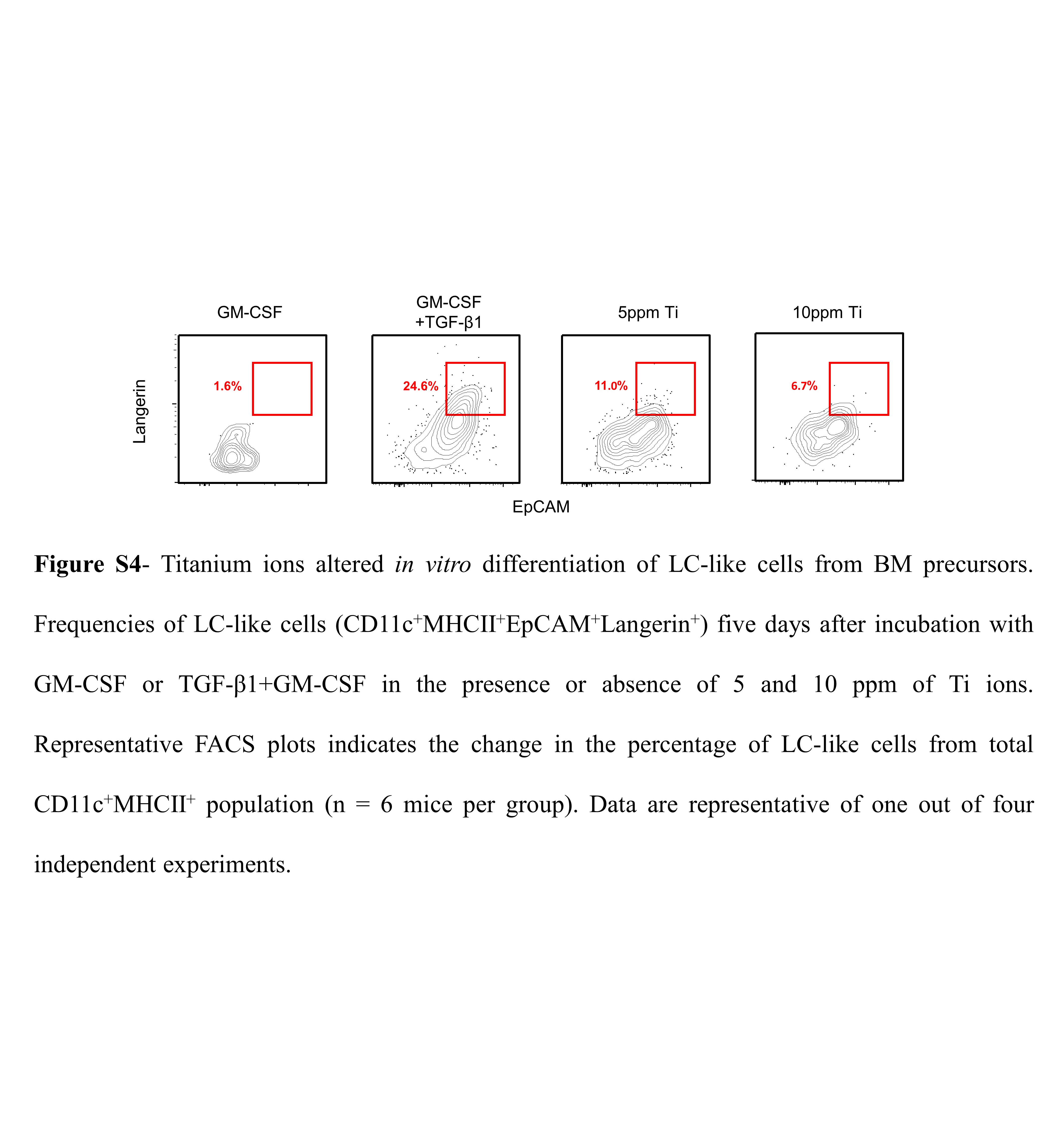

Supplement: Supplementary file 4 [file image_4.jpeg]

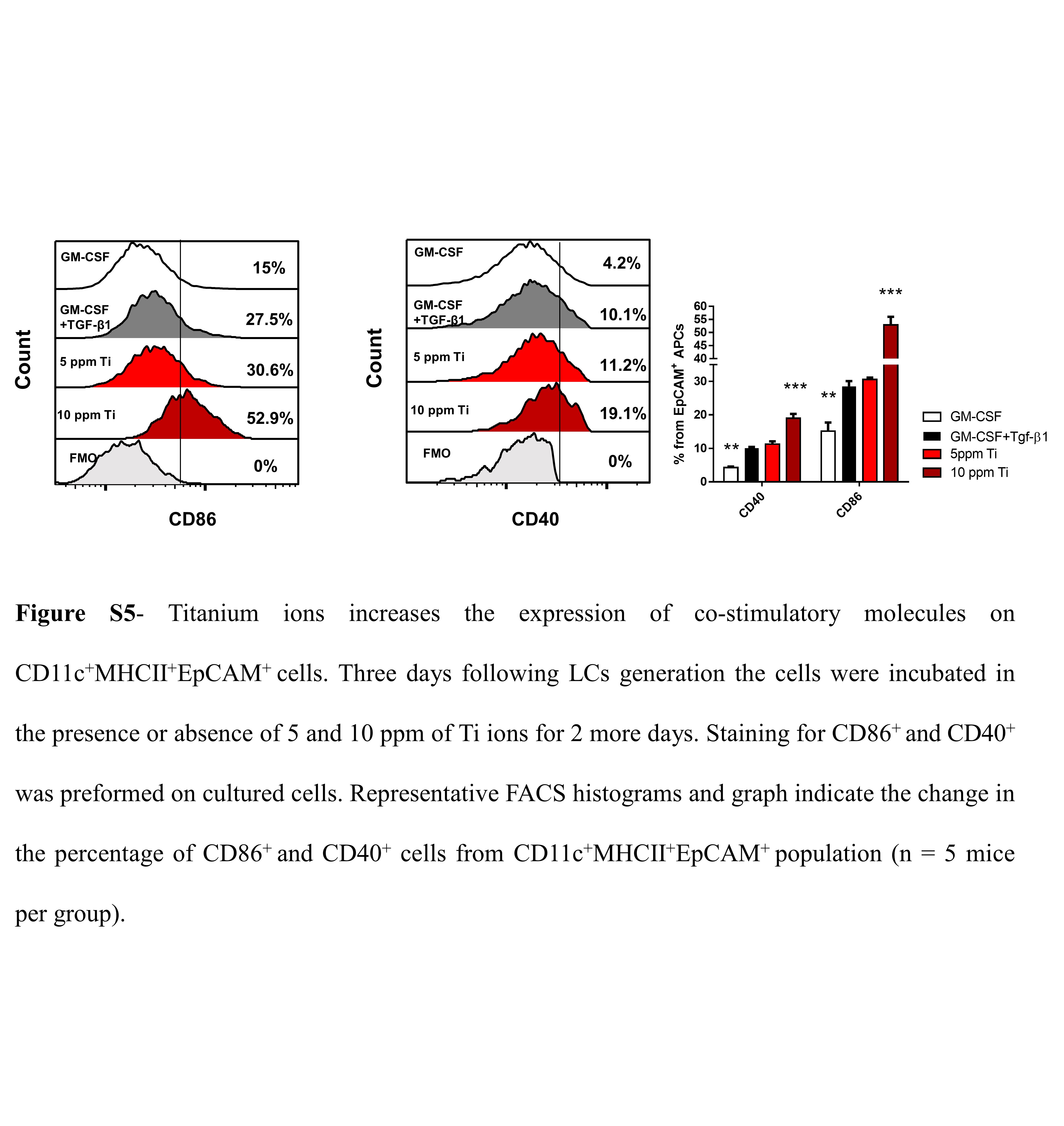

Supplement: Supplementary file 5 [file Image_5.jpg]
